# Effect of Long-Term Fertilization on Aggregate Size Distribution and Nutrient Accumulation in Aeolian Sandy Soil

**DOI:** 10.3390/plants11070909

**Published:** 2022-03-29

**Authors:** Ziru Niu, Fangjiao An, Yongzhong Su, Tingna Liu, Rong Yang, Zeyu Du, Shiyang Chen

**Affiliations:** 1 Linze Inlan River Basin Research Station, Chinese Ecosystem Research Network, Key Laboratory of Eco-Hydrology of Inland River Basin, Northwest Institute of Eco-Environment and Resources, Chinese Academy of Sciences, Lanzhou 730000, China; niuziru12345@163.com (Z.N.); anfj@lzb.ac.cn (F.A.); liutn@lzb.ac.cn (T.L.); yangrong@lzb.ac.cn (R.Y.); duzeyu@nieer.ac.cn (Z.D.); chenshiyang@nieer.ac.cn (S.C.); 2 University of Chinese Academy of Sciences, Beijing 100049, China

**Keywords:** long-term fertilization, aggregate size distribution, nutrient accumulation, aeolian sandy soil, water-stable aggregates, organic carbon

## Abstract

Soil aggregates are the material basis of soil structure and important carriers of nutrients. Long-term application of organic and inorganic fertilizers can affect the composition of soil aggregates to varying degrees, which in turn affects the distribution and storage of soil nutrients. We report the results of a 15-year long-term field-based test of aeolian sandy soil and used the wet sieve method to analyze the stability of water-stable aggregates, as well as the distribution characteristics of nutrients in different particle size aggregates. Our results show that long-term application of organic fertilizer (M3) and combined organic–inorganic treatments (NPK1-M1, NPK1-M2, and NPK1-M3) help to increase the amount of organic carbon, inorganic carbon, and cation exchange in the macro-aggregates, and the improvement rates are 92–103%, 8–28%, and 74–85%, respectively. The organic content of the fertilizers also promotes the formation of macro-aggregates, and the stability of aggregates increase from 0.24 to 0.45. In contrast, the application of inorganic fertilizers (NPK1, NPK2, and NPK3) has no marked effect on the formation and stability of macro-aggregates; the application of inorganic fertilizers can merely maintain the organic carbon content of the soil. Correlation analysis shows that the application of organic fertilizers and chemical (inorganic) fertilizers containing phosphorus and potassium can markedly increase the content and reserves of available phosphorus and potassium across all aggregate sizes, and there is a significant positive correlation between these parameters and the amount of applied fertilizer (*p* < 0.05). Aggregates of various sizes in aeolian sandy soils in arid areas have the potential for greater nutrient storage. Therefore, organic fertilizers can be used in the agricultural production process to improve soil structure and fertility.

## 1. Introduction

In the past half century, due to population increase and food demand, the arid regions of northwest China have experienced large-scale agricultural land development. A large amount of wasteland has been transformed into farmland. The area of oasis farmland has seen a four-fold expansion in the past 60 years [1,2,3]. Land use changes from desert to farmland have resulted in changes to a series of ecological processes, including those relating to water, soil, air, and life in arid areas [4]. For soil, once deserts are transformed into oasis farmland, tillage, irrigation, and fertilization are used to accelerate the process of soil development [5]. In subsequent agricultural use, the evolution of soil properties is strongly influenced by agricultural management measures [6]. The evolution of soil properties affects soil water infiltration, migration, and crop irrigation water requirements, which in turn affects farmland hydrological processes and water management [7,8,9]. The stability of an oasis and the continuous improvement of farmland productivity and irrigation water use efficiency depend on the continuous improvement of soil fertility [10]. Therefore, in the process of oasis conversion from deserts to farmland, changes in soil development processes and nutrient content can not only indicate the evolution of the structure and function of the oasis ecosystem, and the evolution of agricultural productivity, but are also important indicators for evaluating land use and farmland management measures.

Soil structure plays an important role in the biological and physical processes taking place within farmland soil [11,12,13]. Soil aggregates are the basic unit of soil structure [14], comprising a heterogeneous combination of minerals and organic particles; their size, shape, and stability directly affect soil aeration and water availability [15]. The physical, chemical, and biological properties of soil are closely related to crop growth [16]. Soil organic carbon is a core element that affects soil quality and functional performance; it plays an important role in enhancing the aggregation of soil particles and promoting the formation of aggregate structure [17,18,19,20]. However, in arid regions, inorganic carbon is also an important cementing agent for the formation of aggregates, and its effect on the formation and stability of aggregates cannot be ignored [21]. Soil aggregates are considered to be “resident reservoirs” of soil nutrients, and an increase in their number indicates an increase in soil nutrient storage capacity [22]. It is generally agreed that macro-aggregates are the best carriers of soil organic carbon and nutrients. Large amounts of soil organic carbon and nutrients are sequestered in large aggregates, but this tends to occur only in soils in semi-arid and humid regions [23,24]. In arid areas, due to a dry climate and scarce precipitation, desert soils are often characterized by poor soil structure and low nutrient content; in such cases, the silt + clay fraction may be the main carrier of soil organic carbon and nutrients.

Fertilizer application is the main way to maintain soil fertility. Long-term application of organic and inorganic fertilizers to farmland may affect the regeneration and transformation of soil structure [25]. The application of organic fertilizers and mineral fertilizers can affect the composition of soil aggregates to varying degrees, and at the same time change the habitat of soil organisms, which in turn affects the distribution and availability of soil nutrients [26]. For example, many studies have shown that long-term application of farm manure can increase the content of carbon and nitrogen throughout the soil and change the distribution ratio of soil aggregates. In addition, after adding organic residues to the soil, the amount of carbon sequestered in the soil and the stability of aggregates have been shown to markedly improve [27]. Notably, the application of inorganic fertilizers has had contrasting results on the soil organic carbon content and aggregate distribution. Egodawatta et al. and Ghosh et al. found that the application of inorganic fertilizers to soil promoted the formation of macro-aggregates and increased the organic carbon content in these aggregates [28,29]. However, Bi et al. observed that long-term application of inorganic fertilizers reduced the organic carbon content throughout the soil and reduced the aggregate content [30]. Some studies have found that the application of inorganic fertilizer (NPK) can merely maintain the organic carbon concentration of a soil and has no marked effect on changing the soil organic carbon or macro-aggregate content [31,32,33]. In addition, Gelaw et al. found that the content of organic carbon, total nitrogen, and available phosphorus in soil aggregates mainly depends on clay particles, which may have different distributions in different aggregate sizes [34]. Although the organic carbon and total nitrogen related to soil aggregates have been extensively studied, there are few related studies on available phosphorus, available potassium, and exchangeable cations in soil aggregates in arid areas.

In this study, a long-term fertilization experiment was established in intensively cultivated aeolian sandy soil, where corn–wheat rotation was implemented to monitor changes in plant and soil fertility under the treatment of farmhouse manure and mineral fertilizers. Although the impact of fertilization on the quality of sandy soil has been well documented, previous studies have mainly focused on total soil carbon pools, greenhouse gas emissions, and soil nutrient accumulation [35,36]. Soil organic carbon dynamics, changes in active organic carbon components and carbon stabilization mechanisms under different agricultural management measures have been studied in depth in paddy soils in the south, in black soils in the northeast, in fluvo-aquic soils and cinnamon soils in the North China Plain, and during the drought in northwest China [37,38,39,40]. Studies on desert soils in the region are rarely reported, especially for newly cultivated desert sandy soils, which have extremely low organic matter and nutrient content, and the changes in nutrient content and storage in soil aggregates may respond more rapidly to different types of fertilization, but related research is lacking.

In order to further understand the effects of fertilization on soil nutrients, long-term experiments are required. Therefore, the purpose of this study was to clarify the changes in distribution of the main soil nutrients in aggregates under different long-term fertilization conditions. Accordingly, we put forward the following hypotheses for testing: (i) after a long period of various fertilization measures, the organic carbon and nutrient content throughout the soil will change due to the different fertilization methods; (ii) the variation range of the organic carbon and nutrient reserves in the soil aggregates during various fertilization processes will change due to the size of the aggregates; this is due to differences in the preservation of soil nutrients in different aggregate fractions.

## 2. Results

### 2.1. The Effect of Long-Term Fertilization on the Physical and Chemical Properties of Soil

After 15 years of the combined application of different fertilizers, the soil texture under all treatments changed in comparison to the soil properties at the beginning of the experiment in 2005 (Table 1). The sand content in the soil gradually decreased, and the silt + clay content gradually increased. Organic fertilizer and mixtures of organic and inorganic fertilizers (M3; NPK1-M1; NPK1-M2; NPK1-M3) reduced the soil bulk density, while the application of inorganic fertilizers (NPK1, NPK2, NPK3) had no major effect on the soil bulk density. All long-term fertilization treatments reduced the soil pH. Long-term application of inorganic fertilizers increased the soil organic carbon content by a small margin, while the individual application of organic fertilizers, and combined application of organic and inorganic fertilizers, resulted in a markedly higher soil organic carbon content than that seen in 2005.

Compared with the long-term application of inorganic fertilizers (NPK1, NPK2, NPK3), the application of organic fertilizers alone or the combined application of organic and inorganic fertilizers (M3; NPK1-M1; NPK1-M2; NPK1-M3) increased soil organic carbon, inorganic carbon, total nitrogen, and EC_tot_ content by 72–153%, 8–21%, 7–14%, and 7–14%, respectively. The application of inorganic fertilizer containing phosphorus and potassium or combined organic–inorganic application increased the content of available phosphorus and available potassium in the soil. Compared with NPK1, the content of available phosphorus and available potassium after NPK3 application increased by 232% and 37%, respectively. The content of available phosphorus and available potassium after NPK1-M1 application increased by 266% and 113%, respectively, and the content of available phosphorus and available potassium after NPK1-M3 application increased by 403% and 163%, respectively.

### 2.2. The Effect of Long-Term Fertilization on the Distribution and Stability of Soil Aggregates

Long-term application of inorganic fertilizers (NPK1, NPK2, NPK3) had no marked effect on the size composition of aggregates, while the application of organic fertilizer or combined organic–inorganic application markedly increased the proportion of macro-aggregates (Table 2). Compared with NPK1, M3, NPK1-M1, NPK1-M2, and NPK1-M3 treatments increased the mass fraction of aggregates of each particle size by 152%, 79%, 125%, and 146%, respectively. The mass fraction of silt + clay decreased notably; compared with NPK1, the reduction rates for M3, NPK1-M1, NPK–M2, and NPK–M3 were 27%, 23%, 25%, and 32%, respectively. The MWD after the additional organic fertilizer treatments was markedly higher than that after the treatments with inorganic fertilizer; it showed a maximum value after the NPK1-M3 treatment and decreased in the following order: NPK1-M3 > M3 > NPK1-M2 > NPK1-M1 > NPK1 > NPK3 > NPK2.

### 2.3. Relationship between Soil Organic Carbon, Inorganic Carbon, Total Nitrogen and Cation Exchange Capacity and Aggregate Stability

**Figure 1:**
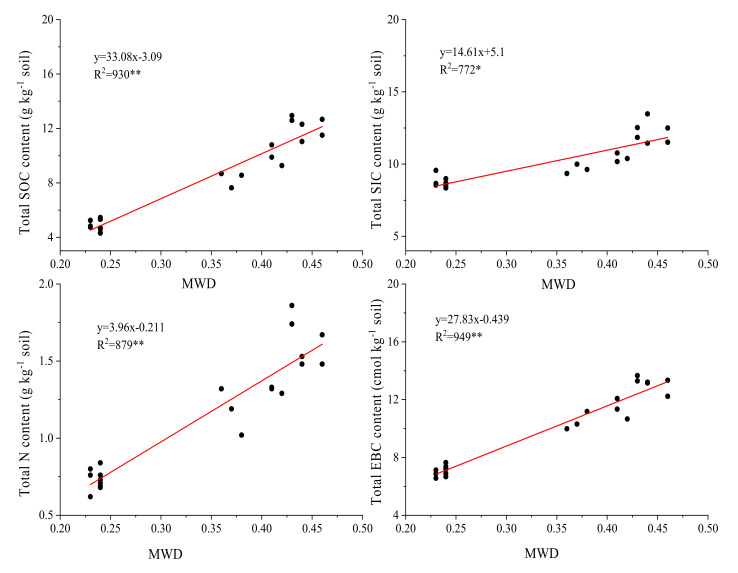
Relationship between total SOC, total SIC, TN, ECtot and aggregate stability in terms of mean weight diameter (MWD). * and ** indicate significance at *p* < 0.05 and *p* < 0.01, respectively.

In each fertilization treatment, the aggregate content of each particle size was positively correlated with soil organic carbon, inorganic carbon, total nitrogen and cation exchange, and reached a significant or extremely significant level (Table 3). The silt + clay content was significantly negatively correlated with soil organic carbon, inorganic carbon, total nitrogen and cation exchange capacity. For macro-aggregates and micro-aggregates, the correlation coefficients between macro-aggregates and each index were relatively high, indicating that macro-aggregates can be used as an important indicator to characterize soil quality. When the content of macro-aggregates in the soil is high, the soil is at a higher fertility level. The correlation analysis between the aggregate stability and soil organic carbon, inorganic carbon, total nitrogen and cation exchange capacity in each fertilization treatment was carried out (Figure 1). It was positively correlated with the cation exchange capacity, and reached a significant or extremely significant level, and the order of the correlation was cation exchange capacity > organic carbon > total nitrogen > inorganic carbon.

### 2.4. The Effect of Long-Term Fertilization on the Content of Nutrients in Soil Aggregates

**Figure 2:**
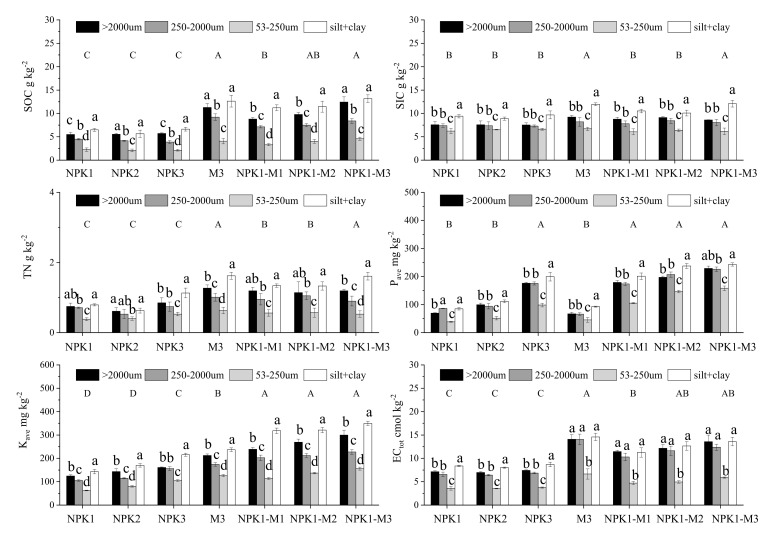
The effect of long-term fertilization on the SOC, SIC, TN, P_ave_, K_ave_ and EC_tot_ content of aggregate fractions. Bars represent the mean + standard error (*n* = 3). Different letters indicate significant differences among the treatments (*p* < 0.05).

The nutrient content in the aggregates of each particle size showed a V-shaped distribution law as a whole (Figure 2). The >2000 μm macro-aggregates and silt + clay organic carbon change intervals are divided into 5.51–12.43 g·kg^−1^ and 6.47–13.15 g·kg^−1^, respectively, which are significantly higher (*p* < 0.05) than the organic carbon content of the 2000–250 μm macro-aggregates and 250–53 μm micro-aggregates (3.87–9.19 g·kg^−1^ and 2.08–4.58 g·kg^−1^, respectively). The distributions of inorganic carbon, total nitrogen, quick-acting phosphorus, quick-acting potassium, and cation exchange capacity across each particle size agglomerate are similar to the distribution of organic carbon, all showing characteristic V-shaped distributions.

Compared with NPK1, the application of a large amount of inorganic fertilizer had no marked effect on the content of organic carbon, inorganic carbon, total nitrogen, and cation exchange capacity across all sizes of aggregates, while the individual application of organic fertilizer and combined organic–inorganic treatments markedly increased the content of organic carbon, inorganic carbon, total nitrogen, and cation exchange in all fractions. Among them, the organic carbon content in granular aggregates increased most notably, by 119–136%, whereas the inorganic carbon content in the granular aggregates showed the smallest increase of 14–23%.

The application of inorganic fertilizer containing phosphorus and potassium, or combined organic–inorganic treatments, increased the content of available phosphorus and potassium in aggregates of each particle size. Compared with NPK1, the content of available phosphorus and potassium after NPK3 application increased by 104–156% and 29–67%, respectively; the content of available phosphorus and potassium after NPK1-MI treatment increased by 102–174% and 82–122%, respectively; and the content of available phosphorus and potassium after NPK1-M3 treatment increased by 162–362% and 115–149%, respectively.

### 2.5. The Effect of Long-Term Fertilization on Nutrient Reserves in Soil Aggregates

**Figure 3:**
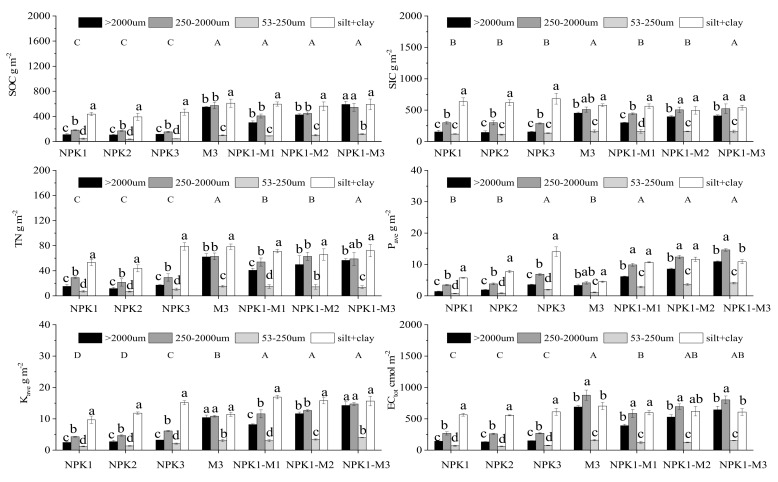
The effect of long-term fertilization on the SOC, SIC, TN, P_ave_, K_ave_ and EC_tot_ stocks within aggregates fractions. Bars represent the mean + standard error (*n* = 3). Different letters indicate significant differences among the treatments (*p* < 0.05).

Among the aggregates treated with inorganic fertilizer, the nutrient reserves in each fraction showed an M-shaped distribution pattern as a whole (Figure 3). The 2000–250 μm macro-aggregates and silt + clay fractions were the main carriers of nutrients; here, the reserves of organic carbon, inorganic carbon, total nitrogen, available phosphorus, available potassium, and cation exchange capacity ranged from 392 to 467 g·m^−2^, 634 to 681 g·m^−2^, 44 to 79 g·m^−2^, 5.72 to 14.05 g·m^−2^, 9.68 to 15.15 g·m^−2^, and 557 to 611 cmol·m^−2^, respectively. The reserves of nutrients in 250–53 μm micro-aggregates were the lowest; in this fraction, the reserves of organic carbon, inorganic carbon, total nitrogen, available phosphorus, available potassium, and cation exchange capacity ranged from 35 to 43 g·m^−2^, 110 to 133 g·m^−2^, 6.76 to 10.63 g·m^−2^, 0.74 to 1.97 g·m^−2^, and 60 to 75 cmol·m^−2^, respectively.

However, for the application of a single organic fertilizer or combined organic–inorganic application, the nutrient reserves in each size aggregate showed a W-shaped distribution law as a whole. The >2000 μm macro-aggregates and silt + clay fractions were the main carriers of various nutrients, and the combined organic–inorganic treatment markedly increased the nutrient reserves in each particle size. The reserves of organic carbon, inorganic carbon, total nitrogen, available phosphorus, available potassium, and cation exchange capacity after NPK1-M1 treatment increased by 37–175%, 37–98%, 34–171%, 87–343%, 75–229%, and 6–176%, respectively, while after NPK1-M3 treatment they increased by 35–438%, 35–170%, 36–278%, 90–690%, 60–675%, and 8–353%, respectively.

### 2.6. The Relationship between Fertilizer Input and Soil Nutrients Storage

The correlation analysis between the aggregate stability and soil organic carbon, inorganic carbon, total nitrogen and cation exchange capacity in each fertilization treatment was carried out (Table 4). It was positively correlated with the cation exchange capacity, and reached a significant or extremely significant level, and the order of the correlation was cation exchange capacity > organic carbon > total nitrogen > inorganic carbon. There was a significant positive correlation between the input amount of inorganic fertilizer and the available phosphorus and potassium reserves in the aggregates of each particle size. The difference is that the input amount of inorganic fertilizers has a low correlation with the organic carbon, inorganic carbon, total nitrogen and cation reserves in large aggregates (>250 μm), or even negatively correlated. The organic carbon, inorganic and total nitrogen carbon storage in 250–53 μm micro-aggregates and silt + clay had a high correlation with inorganic fertilizer input, and even reached a significant positive correlation, indicating that with the increase in inorganic fertilizer application, the nutrients from macro-aggregates were gradually transferred to micro-aggregates and silt + clay particles.

## 3. Discussion

### 3.1. The Effect of Long-Term Fertilization on the Distribution and Stability of Aeolian Soil Aggregates

Soil aggregates not only affect the supply of water and nutrients needed for plant growth, but also affect material exchange in the soil, microbial activities, and crop root extension processes [41]. Cementitious substances are the basic materials for the formation of soil aggregates, and their mass fraction, distribution, composition characteristics, and mode of action play an important role in the formation and stability of aggregates [42]. It is generally accepted that the dynamics and stability of aggregates depend mainly on the content of organic matter, clay, and oxides in the soil [43,44]. Organic matter binds native soil particles with multivalent cations and clay particles to form composite particles. These composite particles form macro-aggregates through the bonding of particulate organic matter (POM) and other cementing materials [45]. In arid and semi-arid regions, the formation of calcium carbonate is also closely related to the dynamics of soil aggregates. Guo et al. used aggregate analysis experiments and scanning electron microscopy to study the role of calcium carbonate in soils in arid areas and found that a small number of large particles of calcium carbonate act as skeletons and are embedded in macro-aggregates, and most small particles of calcium carbonate adhere to the surface of minerals; these calcium carbonates exert an irreversible cementation effect on the formation of aggregates [46]. Our results show that in all treatments, organic carbon and inorganic carbon in aeolian soil aggregates are mainly distributed in the >2000 μm macro-aggregate and silt + clay fractions, and the concentration of organic carbon and inorganic carbon decreases as the size of the aggregates becomes smaller (Table 1). These results verify that organic carbon and inorganic carbon exist in the soil as the main cementing substances and play a vitally important role in the formation of aeolian sandy soil aggregates. Our previous research has shown that most of the oasis farmland soils that occur on the edge of the middle Hexi Corridor are new sandy soils that were cultivated during irrigation [47]. Through monitoring the physical and chemical properties of farmland soils in a long-term reclamation sequence, we found that this soil has a high sand content and low nutrient content. Even though it is richer in iron oxides than red soil in southern China, it lacks OC, which is a key cementing substance. Consequently, these sandy soils cannot easily form stable aggregates [48]. Oades proposed that micro-aggregates are formed within macro-aggregates, and when the macro-aggregates are broken, the micro-aggregates are released [49]. At the same time, the formation of micro-aggregates is often accompanied by the decomposition of particulate organic matter in macro-aggregates [50]. Therefore, the content of organic carbon and inorganic carbon in micro-aggregates is lower than that in macro-aggregates.

Fertilization is one of the key measures to maintain soil productivity and increase crop yields [51]. Long-term application of different fertilizers may change the soil structure, which in turn leads to the redistribution of soil aggregates (Table 5) [52]. Climate change can affect soil aggregates through changes in temperature and moisture status and dry–wet, freeze–thaw cycles, etc. It can cause the rearrangement of soil particles, and soil management measures (including fertilization) can alter the content of organic carbon in agglomerates, leading to changes in soil aggregates [53]. The MWD is a common indicator reflecting the size distribution of soil aggregates after wet screening. The larger the value, the higher the degree of soil aggregates, the better the structural stability, and the stronger the soil erosion resistance [54]. Correlation analysis shows that the MWD in aeolian sandy soil is positively correlated with the organic carbon, inorganic carbon, total nitrogen, and cation exchange capacity in the soil (Figure 1). Our results show that compared with NPK1, the application of a single organic fertilizer and combined organic–inorganic applications (M3, NPK1-M1, NPK1-M2, NPK1-M3) markedly improved the stability of aggregates. While increasing the mass fraction of >2000 μm and 2000–250 μm macro-aggregates, it also caused a decrease in the mass fraction of silt + clay (Table 1). This shows that long-term application of exogenous organic fertilizers in aeolian sandy soil can increase the degree of soil aggregates and improve the soil structure. This may be due to the input of fresh organic residues contained within the organic fertilizer, which increases the soluble organic carbon content and microbial activity in the soil, resulting in the cementation of micro-aggregates to form macro-aggregates; this increases the content of large aggregates, ultimately increases the degree of aggregation within the soil, and improves the stability of the aggregates (Table 2). Lugato et al. and others have found that the application of organic fertilizer can increase the composition of macro-aggregates, thereby promoting the degree of soil aggregation [55]. Subsequently, Zhang et al. used stable C-isotope tracing technology to prove that adding more easily decomposable new carbon can more effectively sequester soils with high organic matter content [56]. In the medium, there was an increase in the distribution ratio of macro-aggregates, and it is more difficult to accumulate in soils lacking organic matter. Moreover, many studies have shown that the application of organic fertilizer stimulates soil microbial enzyme activity while also providing organic matter to the soil, and this has a beneficial effect on the formation and stability of aggregates [57,58].

Previous studies have shown that the application of inorganic chemical fertilizers can also increase the number of aggregates [59]. Our results are remarkable in this regard; although chemical fertilizers (NPK) were continuously added to aeolian sandy soil for 15 consecutive years, the application of inorganic fertilizers could only maintain the whole soil and organic aggregates. The carbon content and MWD did not change meaningfully. Bhattacharyya et al. found similar results [60]. Studies have shown that adding inorganic fertilizers to soils lacking nutrients can only increase the organic carbon content of silt + clay fractions, neither promoting the degree of soil aggregation nor changing the mass fraction composition of soil aggregates. Zhang et al. and Chivenge et al. found that the application of inorganic fertilizer alone can cause soil acidification and accelerate the mineralization and decomposition of soil organic carbon, thereby reducing the proportion of macro-aggregates [61]. From this point of view, whether organic fertilizer is applied alone over a long time period, or combined with inorganic fertilizers, it will promote the formation of large soil aggregates and promote the beneficial development of the main soil structure. While the single application of inorganic fertilizers may lead to macro-aggregates, the disintegration of the body of the soil and the formation of more small aggregates will reduce the stability of the main soil structure.

### 3.2. The Effect of Long-Term Fertilization on the Nutrient Content in Aeolian Sandy Soil Aggregates

The results of long-term experiments involving soil fertilizers show that the application of organic fertilizers is considered an effective way to increase the organic and nitrogen content of aggregates [62,63,64,65]. The results herein show that, compared with NPK1, there is no substantial difference between the organic carbon and inorganic carbon contents of the aggregates after NPK2 and NPK3 application, while the content of organic carbon and inorganic carbon in the aggregates after NPK1-M1, NPK1-M2, and NPK1-M3 treatments varies with fertilizer. The amount of application increased (*p* < 0.05) (Figure 2 and Figure 3). This may be because organic fertilizers contain a large amount of exogenous organic matter. These organic substances are decomposed and humified to produce a large number of short-chain compounds. These short-chain compounds are mainly polysaccharides, polypeptides, aliphatic compounds, and polycyclic compounds, and these lignin fragments can combine with soil cations and soil mineral particles to form soil aggregates. Li et al. found that within the first month after adding POM to the soil, almost all POM in the aggregates is present in macro-aggregates, instead of being stabilized in the free micro-aggregates or adsorbed to the free silt + clay [66]. Our results show that in the process of applying organic fertilizer and mixed organic–inorganic application, the SOC and SIC concentrations in each aggregate fraction increase. Wang et al. and Guo et al. conducted research on farmland soil carbon in Xinjiang and Gansu, which showed that soil inorganic carbon and organic carbon are positively correlated [67,68]. The research shows that due to the driving effect of SOC, the application of organic fertilizer can markedly activate soil carbonates, as in the case of piles of artificial soil.

The growth and decline trends of soil organic carbon and total nitrogen are often consistent [69]. Our results also show that the content and distribution of total nitrogen in aggregates are similar to those of organic carbon. Compared with NPK1, there is a marked difference in total carbon in aggregates after NPK2 and NPK3 application, while the total carbon content in aggregates after the NPK1-M1, NPK1-M2, and NPK1-M3 treatments increases with an increase in fertilizer application. This may be because the chemical fertilizers used in this study are inorganic nitrogen fertilizers. This part of inorganic nitrogen fertilizers rarely accumulates in soil organic matter, especially in the smaller aggregates without physical protection, which are more likely to be leached. Rasmussen et al. found that the effect of chemical fertilizer on soil nitrogen content is determined by its net residue in the soil [70]. Chemical fertilizer has neither significant net stimulation nor significant net residue on soil nitrogen mineralization, so its role in increasing soil total nitrogen content is not obvious. Unlike nitrogen fertilizers, most organic fertilizers have obvious net residues in the soil, so they help to increase the total nitrogen content of the soil. In addition, Ostrowska and Porebska also found that large amounts of nitrogen fertilizer can increase the amount of crop stubble and root exudates, that is, increase the amount of organic nitrogen returned to the soil, making it easier to recycle than the original soil organic nitrogen [71].

Soil cation exchange capacity is closely related to the formation and stability of soil aggregates [72]. A study by Goladi found that many studies have shown that the amount of organic carbon greatly affects the value of soil cation exchange [73]. Humus has a large specific surface area and a large number of functional groups that can be hydrolyzed to generate negative charges, which helps determine the amount of cation exchange. Our results show that the application of inorganic fertilizer has no notable effect on the cation exchange capacity of aggregates of each particle size, while the application of a single organic fertilizer or combined organic–inorganic application markedly increases the cation exchange capacity of the aggregates. This may be because the amount of cation exchange in the soil is mainly determined by the nature of soil colloids. The manure in the organic fertilizer and combined organic–inorganic treatments in this study contains a high content of humus, resulting in an increased content of organic matter. The amount of cation substitution is also large in manure, which can improve the ability of soil to retain water and fertilizer. Liu et al. analyzed the nutrient content in aeolian sandy soil aggregates and found that the cation exchange capacity of 250–53 μm micro-aggregates was significantly lower than that of other particle sizes, but the content of a given particle size did not differ between different treatments [74]. Notably, this shows that the cation exchange capacity of the soil is related to the aggregation capacity of the aggregates. The higher the cation exchange capacity, the stronger the aggregation capacity of the soil. This study found that the cation exchange capacity of different treatments differed markedly among particle sizes, and the cation exchange capacity of each treatment was the lowest among the 250–53 μm micro-aggregates. This may be caused by the level of organic matter content in different size aggregates. The studies of Adesodun et al. and Emadi et al. also showed that soil exchangeable cations were mainly concentrated in macro-aggregates and silt + clay fractions in uncultivated soil [75,76]. However, during the cultivation process, as cultivation time increased, the exchangeable cation content of macro-aggregates gradually decreased and that of the silt + clay fractions gradually increased.

Phosphorus and potassium play important roles as plant macronutrient elements and in the growth and development of vegetation. The content of soil phosphorus is relatively low, and the effective phosphorus pool of farmland soil is mainly the result of the long-term application of fertilizer phosphorus [77]. Our results show that the long-term application of phosphorus and potassium-containing chemical fertilizers and organic fertilizers can increase the content of available phosphorus and available potassium in soil aggregates to varying degrees; when chemical fertilizers are combined with organic fertilizers, the quick-acting effects in the aggregates of each particle size lead to the content of available phosphorus and available potassium increasing more markedly. Elliott et al. found similar results in a field experiment using fresh poultry manure to treat soil [78]. The applied phosphorus is preferentially adsorbed by clay particles in the soil, and then this phosphorus will be selectively adsorbed to aggregates of different sizes. Sauer et al. found that the continuous application of organic fertilizers can significantly increase the available potassium and slow-acting potassium content of soil [79]. Moreover, the content of available phosphorus and available potassium is highest in macro-aggregates and lowest in micro-aggregates, which also indicates that the aggregates have the function of coating available potassium and available phosphorus and have the ability to maintain nutrients.

### 3.3. The Effect of Long-Term Fertilization on Nutrient Reserves in Soil Aggregates

The profit and loss of the nutrient balance of the farmland ecosystem is the fundamental factor that determines the growth and decline of the soil nutrient level. Therefore, the change in farmland nutrient reserves is the fundamental basis for judging the development trend of the soil nutrient level. Considering that soil nutrients will markedly affect the stability and fertility of soil aggregates, maintaining nutrient storage is of great importance for the sustainable use of soil resources [80]. Correlation analysis was conducted between the nutrient reserves of the whole soil and aggregates of each particle size, and the input amount of inorganic fertilizer and combined organic–inorganic application. The results show (Table 4) that the application amount of inorganic fertilizer was related to the available phosphorus both in the whole soil and in aggregates of each particle size. The amount of inorganic fertilizer applied also shows a significant correlation with available potassium, but no clear correlation exists with organic carbon, inorganic carbon, total nitrogen, or cation exchange capacity. The difference is that, in addition to the silt + clay fractions, the combined organic–inorganic application is significantly or extremely significantly related to the nutrient reserves of the whole soil and various particle size aggregates. In this study, after 15 consecutive years of fertilization, the carbon and nutrient reserves in macro-aggregates and silt + clay show a greater contribution to the carbon and nutrients in the whole soil than those in other size fractions, which indicates that macro-aggregates and silt + clay are the main SOC and nutrient carrying components of the soil. Compared with micro-aggregates, macro-aggregates have a higher organic carbon and nutrient content, indicating that the macro-aggregates have a stronger fertility retention effect, which is also the reason why macro-aggregates can store more soil nutrients. However, the application of organic fertilizers generated more macro-aggregates in the soil, and the nutrient reserves in the soil aggregates were mainly affected by changing the distribution of soil aggregates of different sizes. Although the storage of nutrients in the soil is mainly reflected in macro-aggregates, the increase in nutrient reserves in micro-aggregates was higher than that in macro-aggregates, which indicates that among the various particle size aggregates of the soil, micro-aggregates have a larger potential to store nutrients. Therefore, the organic carbon content of micro-aggregates can be used as a potential indicator to evaluate the effect of the long-term combined application of different fertilizers on soil organic carbon sequestration in aeolian sandy soils.

In general, >2000 μm macro-aggregates in the soil are the main component that affects soil nutrient and structural stability, and long-term application of organic fertilizer or combined organic–inorganic treatments can promote an increase in the proportion of macro-aggregates and improvement in the internal nutrient content of soil aggregates; in turn, this improves the soil structure, increases the carbon sequestration potential, and improves soil fertility.

### 3.4. Practical Implications of This Study

Aeolian sandy soil is a desert soil with high carbonate (inorganic carbon) content in arid inland areas, and inorganic carbon and organic carbon play an important role in cementing the formation of soil aggregates. Previous studies have mainly focused on the effect of fertilization on the content of organic carbon in aggregates, while in arid regions, there are not many reports on the effects of fertilization on the contents of inorganic carbon, total nitrogen, available phosphorus, potassium, and cation exchange capacity in aggregates. We explain the effects of the application of organic and inorganic fertilizers on the storage of various nutrients in aeolian sandy soils from the perspective of aggregates.

Combined with the results obtained in this study, future work should make full use of long-term positioning experiments, use solid-CPMAS 13C NMR, stable isotopes and synchrotron radiation and other modern technical means, and go deep into the molecular level to explore the organic carbon and minerals in aeolian sand aggregates. The mechanism of cementation between the two, and the protection mechanism of organic carbon in aeolian sandy soil, is systematically and comprehensively studied.

## 4. Materials and Methods

### 4.1. Study Site

**Figure 4:**
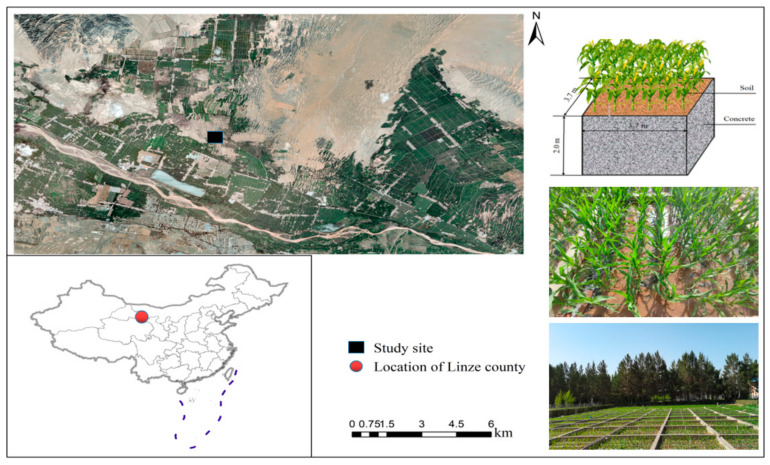
Location of the study site.

All experiments were performed in an oasis on the northern edge of Linze, located in the center of the Hexi Corridor in Gansu (Figure 4). This region is a newly reclaimed oasis that recently underwent transformation from an old oasis into a desert. In the past 30 years, the area of oasis cultivated land has increased from 3636 to 4798 km^2^. The oasis is surrounded by deserts, including the Gobi Desert. It has a typical desert climate, according to the Köppen climatic classification system, and is characterized by cold winters and dry hot summers, with an average annual precipitation of 116.8 mm, an annual evaporation of 2390 mm, an average annual temperature of 7.6 °C, and an annual frost-free period of 165 days. Due to long-term aeolian sand invasion and deposition, aeolian sand is formed in the northern part of the oasis edge, which had a soil type that was identified as an AridiSandic Primosol by the Chinese Soil Taxonomy classification systems [81] and is equivalent to the Aridic Ustipsamments soil in the USDA Soil Taxonomy classification systems [82]. Since the oasis edge desertification control was carried out in the late 1960s, the sandy land has been leveled and gradually reclaimed, forming sandy irrigated farmland with different reclamation time series. Farmland irrigation water mainly depends on Heihe River water and groundwater irrigation. In recent years, the area has mainly grown seed corn and field corn, using traditional tillage and plastic film mulching. The planting period is from March to September. The number of irrigation times during the corn growth period varies from 6 to 11 times due to differences in soil texture. Due to the low fertility of the sandy soil and the poor performance of water and fertilizer retention, it is the area with the largest water consumption and fertilizer application for oasis irrigation in arid areas. The experiment was arranged at the Linze Inland River Basin Research Station of China Ecosystem Research Network (39.24′ N, 100.21′ E, 1350 m above sea level). The test site was flattened by sand dunes in 1975 and fruit trees were subsequently planted. These fruit trees were felled in 2000 and the land was later reclaimed for use as irrigated farmland. The soil is a newly formed sandy soil and the sand content within the 0–100 cm soil layer exceeds 80%.

### 4.2. Experiment Design

In 2000, sand-rich land left behind in the study area after the felling of fruit trees was converted into a test field composed of 4 m × 4 m plots. The soil layer between the plots was separated by waterproof materials to a depth of 0–100 cm, and the ground surface was lined with a layer of concrete with a thickness of 0–20 cm. The uppermost 0–20 cm soil layer became mixed with the clay soil from the old oasis, and the perennial desert plant *Ephedraceae* was planted. The long-term fertilizer experiment subsequently began in this region in 2005. Before the start of the experiment, the soil particle size distribution in the 0–20 cm soil layer was as follows: 65.8%, sand, 20.1% silt, 14.1% clay, with OC and total nitrogen contents of 4.48 and 0.66 g·kg^−1^, respectively. The soil layer had a pH of 8.9.

The experiment involved nine treatments, which were arranged in random blocks and repeated three times. The seven treatments utilized in this study (Table 6) include: NPK1 (low nitrogen, phosphorus, and potassium contents), NPK2 (medium nitrogen, phosphorus, and potassium contents), NPK3 (high nitrogen, phosphorus, and potassium contents), M3 (high organic fertilizer content), NPK1M1 (high nitrogen, phosphorus, and potassium contents, but low organic fertilizer content), NPK1M2 (high nitrogen, phosphorus, and potassium contents, and a medium organic fertilizer content), and NPK1M3 (low nitrogen, phosphorus, and potassium contents, but high organic fertilizer content). Due to the extremely low nutrient content in the sandy soil, the yield of the crop in the control treatment was close to 0, such that the control treatment was discarded in this experiment [83]. The experiment employed a corn–corn–soybean rotation with conventional cultivation. All harvested biomass was removed from the plot during each cycle; consequently, little to no crop residue was returned to the land, and the amount of stubble left in the field was negligible.

### 4.3. Sample Collection and Aggregate Analysis

Sampling was conducted in the field after the corn harvest in September 2020. Three samples were randomly collected from each fertilization treatment plot. The sampling depth was 0–20 cm, soil samples were collected intact, and 3 replicates were taken for each fertilization treatment. We were careful to avoid damaging the soil structure during sampling. Soil samples were packed in a hard aluminum box and transferred back to the laboratory, where the undisturbed soil samples were naturally air-dried indoors. When the soil moisture content reached the plastic limit, the large soil block was broken by hand along natural fractures. The surface was gently broken, gravel and plant residues were removed, and then the soil was passed through an 8 mm soil sieve. After the sample was air-dried, it was used for the separation of water-stable aggregate fractions for later analysis.

The soil aggregates were physically grouped according to the method reported in Six et al. [84]. The operation process was as follows: 100 g of the naturally dried soil sample was weighed, quickly immersed in deionized water, and then placed on a 2000 μm sieve (after being fully soaked). After 3 min of infiltration, the sample was manually shaken up and down 50 times with an amplitude of 3 cm at a constant speed; this process lasted 2 min. The 2000 and 250 μm sieves were slowly rinsed with deionized water to ensure that micro-aggregates were completely flushed above the 53 μm sieve; the soil samples remaining on the 2000, 250, and 53 μm sieves were then collected. The silt + clay fractions passed through the 53 μm sieve and were collected in a pre-prepared large beaker together with the deionized water stream. The silt + clay fractions were taken out and placed in a centrifuge tube, then centrifuged at 5000 rpm for 30 min at 4 ℃. Hence, the above rigorous wet sieving process obtained the following soil aggregate fractions: >2000 μm and 2000–250 μm macro-aggregates, 53–250 μm micro-aggregates, and <53 μm silt + clay. Because our samples contained a large amount of sand, it was necessary to correct for the sand content in the macro-aggregates [85]. This was done by weighing the quantitatively dried macro-aggregates and placing them on the 53 μm sieve. We put the glass ball into the glass ball while gently shaking the sieve; a constant flow of deionized water was used to slowly rinse through the 53 μm sieve, then the sand left on the sieve was dried and weighed to obtain the sand content of the macro-aggregates. After the above separation process, all the aggregate fractions were dried at 60 ℃ and weighed.

### 4.4. Measurement of Soil Physical and Chemical Properties

The soil moisture content was measured after the soil samples were dried at 105 °C. The soil pH and electrical conductivity (EC) were measured using a glass electrode with a soil-to-water ratio of 1:2.5 (*m*/*v*) [86]. The soil particle size distribution was determined using the pipette method in a sedimentation cylinder, using sodium hexametaphosphate as the dispersing agent [87]. The potassium dichromate oxidation method and ferrous ammonium sulfate titration method were used to determine the content of organic matter in the original soil and aggregates [88]. The organic carbon content is equal to the organic matter concentration divided by 1.72; the total nitrogen and total carbon contents of the soil were determined using an elemental analyzer (*Elementar vario MACRO cube, Germany*). The soil inorganic carbon (SIC) content was calculated as the total carbon content minus the soil organic carbon (SOC) content. Available phosphorus and available potassium were extracted using the sodium bicarbonate–molybdenum antimony colorimetric method and ammonium acetate extraction-flame photometry (*722N, Wanli Instrument Strength Factory, China*) [89], and exchangeable cations were measured using the ammonium acetate replacement method [90].

### 4.5. Statistics and Calculations

The average mass diameter of soil aggregates (*MWD*) was calculated as follows:

(1) MWD=∑Xj⋅Wj

where *Xi* is the average diameter of aggregates in any level range (mm), and *Wi* is the percentage of aggregates corresponding to *Xi* [91].

Based on the method established by Eynard et al., SOC storage (SOCS, g·m^−2^) in the bulk soil was calculated as follows:

(2) SOCS=∑(Mi×SOCi)×BD×H×10

where *Mi* denotes the aggregate proportion at the *i*th size in the bulk soil (%); *SOCi* is the content of SOC in aggregates at the *i*th size (g·kg^−1^); *BD* represents the bulk density in the bulk soil (g·cm^−3^); and *H* refers to the soil thickness (cm) [92]. Similarly, nutrient storages in the bulk soil were also obtained.

Microsoft Excel 2010 and Origin 2018 were used to process statistical data and graphics. Data are expressed in the form of mean ± standard deviation; the *SPSS* software package was used to evaluate the significance of the difference between different treatments via one-way analysis of variance (ANOVA; α = 0.05), while the multiple comparison test of the means used the least significant extreme difference method (LSD). For data that had passed the normality test, the Pearson method was used to analyze the correlation of the results.

## 5. Conclusions

Long-term application of different fertilizers affected the distribution of aggregates of various particle sizes in aeolian sandy soil. The application of organic fertilizer and mixtures of organic–inorganic fertilizers can increase the content of macro-aggregates in the soil and improve the stability of the aggregates. The application of inorganic fertilizer can only maintain the degree of soil aggregates. Applying organic fertilizer alone or in combination with inorganic fertilizer increased the content of organic carbon, inorganic carbon, total nitrogen, available phosphorus, available potassium, and cation exchange capacity in each particle size aggregate of the soil. Compared with macro-aggregates, the nutrient content in micro-aggregates increased markedly. The application of inorganic fertilizer containing phosphorus and potassium only increased the content of available phosphorus and potassium in each particle size aggregate and had no notable effect on the content of organic carbon, inorganic carbon, total nitrogen, or cation exchange capacity. Correlation analysis results show that the stability of the soil structure and nutrient reserves in the aggregates are significantly positively correlated with the application amount of organic fertilizer (*p* < 0.05). Therefore, organic fertilizer can be applied in the agricultural production process to improve soil structure and fertility.

## Figures and Tables

**Table 1:** Effects of different fertilization types on the physical and chemical properties of soil.

Treatments	Chemical Fertilizer							
	2005	NPK1	NPK1	NPK3	M3	NPK1-M1	NPK1-M2	NPK1-M3
BD	1.48 ^a^	1.43 ± 0.03 ^bc^	1.44 ± 0.02 ^b^	1.43 ± 0.03 ^bc^	1.40 ± 0.03 ^bc^	1.38 ± 0.02 ^c^	1.38 ± 0.02 ^c^	1.39 ± 0.03 ^bc^
Sand (%)	65.8 ^a^	57.15 ± 1.38 ^b^	56.34 ± 1.78 ^bc^	55.62 ± 2.35 ^bcd^	53.23 ± 1.19 ^cd^	52.32 ± 1.02 ^d^	53.01 ± 2.04 ^cd^	54.81 ± 1.29 ^c^
Silt (%)	20.1 ^b^	27.72 ± 1.34 ^a^	28.17 ± 1.36 ^a^	28.13 ± 0.67 ^a^	28.45 ± 1.74 ^a^	29.36 ± 0.59 ^a^	28.63 ± 1.67 ^a^	27.92 ± 1.37 ^a^
Clay (%)	14.1 ^d^	15.13 ± 0.60 ^c^	15.49 ± 0.78 ^c^	16.25 ± 1.56 ^bc^	18.32 ± 0.54 ^ab^	18.4 ± 0.53 ^a^	18.36 ± 1.36 ^ab^	17.27 ± 0.72 ^ab^
pH	8.9 ^a^	8.62 ± 0.05 ^bc^	8.67 ± 0.04 ^b^	8.45 ± 0.03 ^d^	8.60 ± 0.06 ^bc^	8.46 ± 0.03 ^d^	8.53 ± 0.06 ^cd^	8.53 ± 0.09 ^cd^
EC (μs·cm^−1^)		237 ± 6.7 ^d^	226 ± 9.5 ^d^	241 ± 8.5 ^d^	343 ± 14.1 ^a^	285 ± 11.0 ^c^	306 ± 7.8 ^b^	346 ± 9.7 ^a^
SOC (g·kg^−2^)	4.48 ^e^	4.54 ± 0.19 ^e^	4.46 ± 0.25 ^e^	5.17 ± 0.32 ^d^	12.74 ± 0.66 ^a^	8.29 ± 0.57 ^c^	9.98 ± 0.76 ^b^	11.61 ± 0.64 ^a^
SIC (g·kg^−2^)		8.66 ± 0.30 ^d^	8.53 ± 0.71 ^d^	8.92 ± 0.56 ^cd^	11.97 ± 0.52 ^a^	9.66 ± 0.32 ^c^	10.44 ± 0.30 ^b^	12.47 ± 1.01 ^a^
TN (g·kg^−2^)	0.66	0.75 ± 0.08 ^d^	0.68 ± 0.05 ^d^	0.77 ± 0.02 ^d^	1.76 ± 0.10 ^a^	1.18 ± 0.15 ^c^	1.31 ± 0.02 ^c^	1.5 ± 0.03 ^b^
P_ave_ (mg·kg^−2^)		50.56 ± 4.61 ^g^	109.54 ± 9.23 ^e^	167.61 ± 3.96 ^d^	80.55 ± 6.75 ^f^	184.91 ± 4.69 ^c^	208.75 ± 5.18 ^b^	254.41 ± 10.40 ^a^
K_ave_ (mg·kg^−2^)		106.41 ± 7.30 ^f^	121.51 ± 6.96 ^e^	145.96 ± 9.75 ^d^	233.06 ± 8.43 ^c^	226.88 ± 14.47 ^c^	253.54 ± 9.38 ^b^	280.12 ± 5.92 ^a^
EC_tot_ (cmol·kg^−2^)		7.02 ± 0.30 ^c^	7.29 ± 0.39 ^c^	6.86 ± 0.29 ^c^	13.43 ± 0.20 ^a^	10.49 ± 0.62 ^b^	11.36 ± 0.71 ^b^	12.86 ± 0.55 ^a^

BD: soil bulk density; EC: electrical conductivity; SOC: soil organic carbon; SIC: soil inorganic carbon; TN: total nitrogen; P_ave_: available phosphorus; K_ave_: available potassium; EC_tot_: the exchangeable cation. The values are the average of the values obtained from three replicate samples ± one standard deviation. Where values are presented with different lowercase letters, the difference between them is statistically significant (*p* < 0.05).

**Table 2:** Effect of different fertilization types on the distribution and stability of aggregates.

Treatment	Macroaggregate (>2000 µm)	Macroaggregate (2000–250 µm)	Microaggregate (250–53 µm)	Silt + Clay Fraction (<53 µm)	The Recovery Ratio (%)	MWD
NPK1	6.95 ± 0.65 ^d^	14.16 ± 0.78 ^c^	6.69 ± 0.51 ^bc^	23.56 ± 1.40 ^a^	51.36	0.24 ± 0.01 ^d^
NPK1	6.69 ± 0.41 ^d^	14.27 ± 0.34 ^c^	5.94 ± 0.37 ^c^	24.47 ± 1.32 ^a^	50.37	0.23 ± 0.01 ^d^
NPK3	7.39 ± 0.53 ^d^	13.71 ± 0.32 ^c^	7.00 ± 0.57 ^b^	24.52 ± 1.25 ^a^	52.62	0.24 ± 0.01 ^d^
M3	17.53 ± 1.07 ^a^	22.33 ± 0.91 ^ab^	8.74 ± 0.79 ^a^	17.30 ± 0.59 ^c^	65.90	0.44 ± 0.02 ^ab^
NPK1-M1	12.42 ± 0.67 ^c^	20.68 ± 1.16 ^b^	9.66 ± 0.80 ^a^	19.85 ± 0.86 ^b^	62.61	0.37 ± 0.02 ^c^
NPK1-M2	15.61 ± 0.25 ^b^	21.51 ± 0.49 ^b^	9.02 ± 0.67 ^a^	17.66 ± 1.07 ^c^	63.80	0.41 ± 0.01 ^b^
NPK1-M3	17.11 ± 0.61 ^a^	23.33 ± 1.28 ^a^	9.28 ± 0.2 ^a^	16.11 ± 1.32 ^c^	65.83	0.45 ± 0.02 ^a^

MWD: the mean weight diameter. Values are the average of values from three replicate samples ± one standard deviation. Different letters indicate significant differences (*p* < 0.05) among the different aggregate fractions.

**Table 3:** Correlation between aggregates and different indexes.

Item	Aggregate Size			
	>2000 µm	2000–250 µm	250–53 µm	Silt + Clay
Total SOC (g·kg^−2^)	0.977 **	0.930 **	0.768 **	−0.921 **
Total SIC (g·kg^−2^)	0.905 **	0.844 **	0.664 *	−0.842 ***
Total N (g·kg^−2^)	0.955 **	0.898 **	0.751 **	−0.885 **
Total EBC (cmol·kg^−2^)	0.982 **	0.946 **	0.790 **	−0.929 **

* means significant correlation (*p* < 0.05), ** means extremely significant correlation (*p* < 0.01).

**Table 4:** Relationship between fertilizer input and soil nutrient stocks within aggregate fractions under 15 years of long-term fertilization.

	Item	Bulk Soil	Aggregate Size			
			>2000 µm	2000–250 µm	250–53 µm	Silt + Clay
	SOC (g·m^−2^)					
Inorganic fertilizer input		0.303	−0.880 **	−0.003	0.328	0.328
Combined organic-inorganic fertilizer input		0.932 **	0.969 **	0.834 **	0.879 **	−0.044
	SIC (g·m^−2^)					
Inorganic fertilizer input		0.353	0.043	−0.256	0.617	0.354
Combined organic-inorganic fertilizer input		0.896 **	0.898 **	0.614	−0.057	−0.183
	TN (g·m^−2^)					
Inorganic fertilizer input		0.207	0.39	0.121	0.722 *	0.755 *
Combined organic-inorganic fertilizer input		0.881 **	0.682 *	0.267	−0.2	0.049
	P_ave_ (g·m^−2^)					
Inorganic fertilizer input		0.987 **	0.9770 **	0.941 **	0.942 **	0.965 **
Combined organic-inorganic fertilizer input		0.969 **	0.996 **	0.981 **	0.898 **	0.106
	K_ave_ (g·m^−2^)					
Inorganic fertilizer input		0.943 **	0.804 **	0.965 **	0.939 **	0.958 **
combined organic-inorganic fertilizer input		0.935 **	0.967 **	0.880 **	0.908 **	−0.482
	EC_tot_ (g·m^−2^)					
Inorganic fertilizer input		−0.251	0.383	0.116	0.391	0.549
combined organic-inorganic fertilizer input		0.886 **	0.951 **	0.882 **	0.658	0.072

SOC: soil organic carbon; SIC: soil inorganic carbon; TN: total nitrogen; P_ave_: available phosphorus; K_ave_: available potassium; EC_tot_: the exchangeable cation. * and ** indicate significance at *p* < 0.05 and *p* < 0.01, respectively.

**Table 5:** Effects of long-term fertilization on aggregate stability and carbon and nitrogen content.

Author	Publication Date	Soil Type	Fertilizer Type	Planting Age	Aggregate Stability	Aggregate Carbon and Nitrogen Content	References
Islam	2021	Sandy loam (Typic Haplustept)	M CM	2	(M) Increase (CM) Increase	(M) The content of organic carbon and total nitrogen in the aggregates of each particle size increased. (CM) The total nitrogen content of organic carbon in aggregates of each particle size increased.	[27]
Ghosh	2018	Inceptisol (Typic Haplustept)	NPK NPKM	44	(NPK) Increase (NPK-M) Increase	(NPK) The content of organic carbon and total nitrogen in the aggregates of each particle size increased. (NPK-M) The content of organic carbon and total nitrogen in the aggregates of each particle size increased.	[29]
Zhang	2016	Calcaric Cambisol (FAO classification)	NPK NPKM NPKS	23	(NPK) Increase (NPK-M) Increase (NPK-S) Increase	(NPK) There was no significant difference in the total nitrogen content of organic carbon in macro-aggregates, while the content of carbon and nitrogen in micro-aggregates increased. (NPK-M) The content of organic carbon and total nitrogen in the aggregates of each particle size increased. (NPK-S) The content of organic carbon and total nitrogen in the aggregates of each particle size increased.	[52]
Zhang	2015	Aquic inceptisol (Typic Haplustept)	NPK CM	20	(NPK) Increase (CM) Increase	(NPK) There was no significant difference in the total nitrogen content of organic carbon in macro-aggregates, while the content of carbon and nitrogen in micro-aggregates increased. (CM) The content of organic carbon and total nitrogen in the aggregates of each particle size increased.	[54]
Zhang	2014	Cambisol	SR M	4	(SR) Increase (M) Increase	(SR) The content of organic carbon and total nitrogen in the aggregates of each particle size increased. (M) The content of organic carbon and total nitrogen in the aggregates of each particle size increased.	[55]
Jiang	2010	Red soil	SR M	18	(SR) Increase (M) Increase	(SR) The content of organic carbon and total nitrogen in the aggregates of each particle size increased. (M) The content of organic carbon and total nitrogen in the aggregates of each particle size increased.	[57]
Bhattacharyya	2010	Sandy loam soil (Typic Haplustept)	NPK NPKM	30	(NPK) constant (NPK-M) Increase	(NPK) There is no significant difference in the total nitrogen content of organic carbon in the aggregates of each particle size. (NPK-M) The content of organic carbon and total nitrogen in the aggregates of each particle size increased.	[58]
Lugato	2010	Sandy soils combine with clay soils	NPK M	48	(NPK) Increase (M) Increase	(NPK) There is no significant difference in the total nitrogen content of organic carbon in the aggregates of each particle size. (NPK-M) The content of organic carbon and total nitrogen in the aggregates of each particle size increased.	[53]
Ours	2020	Aeolian sandy soil (Typic Haplustept)	NPK NPKM	15	(NPK) constant (NPK-M) Increase	(NPK) There is no significant difference in the total nitrogen content of organic carbon in the aggregates of each particle size. (NPK-M) The content of organic carbon and total nitrogen in the aggregates of each particle size increased.	

CM: compost; SR: straw residue; M: manure; NPK: nitrogen, phosphorus, and potassium; NPK-M: nitrogen, phosphorus, and potassium combined with manure; NPK-S: nitrogen, phosphorus, and potassium combined with straw residue.

**Table 6:** Application levels of organic and inorganic fertilizers.

Fertilizer Tipes	Fertilizer Input of Each Season (kg/ha)			
	Manure	N	P_2_O_5_	K_2_O
NPK1	0	150	90	90
NPK2	0	225	135	135
NPK3	0	300	225	225
M3	24,000	0	0	0
M1NPK1	12,000	150	90	90
M2NPK1	18,000	150	90	90
M3NPK1	24,000	150	90	90

1, low fertilizer level; 2, medium fertilizer level; 3, high fertilizer level; N, chemical N fertilizer; P, chemical P fertilizer; K, chemical K fertilizer; M, organic manure. For example, NPK1-M3, low chemical N, P, and K fertilizer levels combined with high organic manure levels.

## Data Availability

Not applicable.
